# Obtaining Glenoid Positioning Data from Scapular Palpable Points In Vitro

**DOI:** 10.1155/2013/391260

**Published:** 2013-04-04

**Authors:** Jordan H. Trafimow, Alexander S. Aruin

**Affiliations:** Department of Physical Therapy (MC 898), University of Illinois at Chicago, 1919 W. Taylor Street, Chicago, IL 60612, USA

## Abstract

Both clinical and biomechanical problems affecting the shoulder joint suggest that investigators should study force transmission into and out from the scapula. To analyze force transmission between the humeral head and the glenoid, one must know the position of the glenoid. Studies have analyzed the position of the scapula from the positions of three palpable points, but the position of the glenoid relative to three palpable points has not been studied. Dry scapulae (*N* = 13) were subjected to X-rays and a critical angle, Θ (which relates the plane determined by the three palpable points on the scapula to a plane containing the glenoid center and the first two palpable points) was calculated. The mean value for Θ was 28.5 ± 5.60 degrees. The obtained Θ allows us to determine the position of the glenoid from three palpable points. This information could be used in calculation of forces across the shoulder joint, which in turn would allow optimizing the choice of strengthening exercises.

## 1. Introduction

Both clinical and biomechanical problems affecting the shoulder joint suggest that investigators should study force transmission into and out from the scapula [1]. One clinical problem is the wearing away of the posterior part of the glenoid in patients who need total shoulder replacement [2]. Another problem is dyskinesis, (e.g., abnormal scapular motion) due to various clinical entities such as internal derangement of the shoulder, acromioclavicular instability, and fractured clavicle [1, 3]. In addition, movements of the scapula are subjected to extensive variations, which in itself influence the interaction of glenoid and humeral head [4]. Some authors postulate that dyskinesis causes swimmer's shoulder and impingement syndrome [5, 6].

The biomechanical problem is finding the location of the glenoid. Since the glenoid is always centered about the humeral head [7], the problem becomes finding two coordinates of the glenoid, for example, Euler angles. To the best of our knowledge, there are no studies dealing with this problem. This is mainly because current techniques lack the ability to locate, in three dimensions, the angle between the glenoid center and the force vector from the humeral head. This angle is needed in the calculation of force transmission across the shoulder and to make more definite the diagnosis of dyskinesia.

 To solve these problems there is a need to define the position of the glenoid. We define the center line as a line running from the point at which the spine and medial border meet (O) through the center of the glenoid (G) to a point midway between the most anterior and the most posterior points on the glenoid rim [8, 9]. Since the scapula rotates around the humeral head [7], the center of the humeral head is also on the center line. Thus, the location of the glenoid could be determined using the center line. 

 There is also a need to obtain three points to determine the location of the scapula. Most of the studies on the scapula in the past have used the vertex of the inferior angle of the scapula (Q) and junction (O) of the scapular spine and the medial border (e.g., [10]) providing two points. The third point is one of various points on the acromion [11]. All the previous points are palpable. However, there is no literature data linking the position of the glenoid to the positions of the three palpable points mentioned previously. A possible way of solving such a problem would be obtaining MRIs with the arm in various positions [4]. The problem is getting the central ray parallel to OQ, the line between the inferior angle (Q) and the junction (O). If the central ray is not perpendicular the angle (our Θ) will be incorrect.

 The aim of the study was to introduce a method of locating the glenoid given three palpable points on the scapula. 

## 2. Methods

### 2.1. Materials and Technique

**Figure 1:**
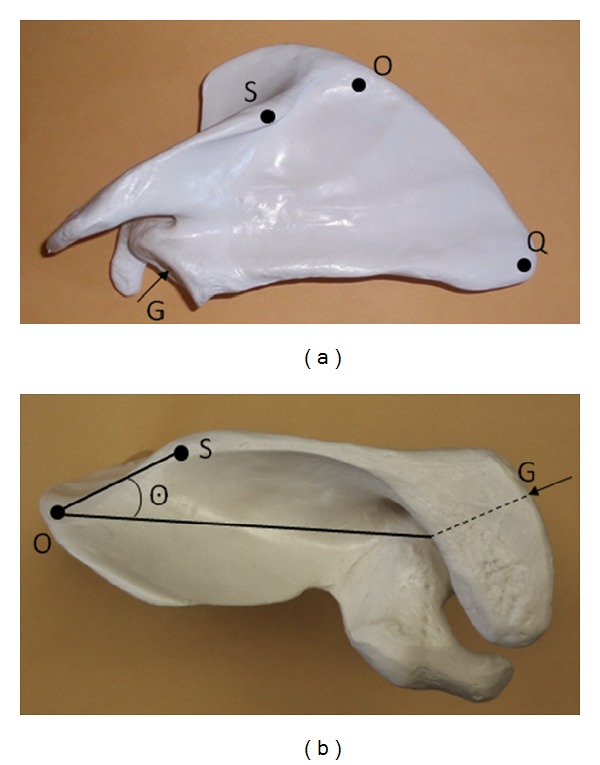
(a) Posterior view of the scapula. The following points are shown: G: center of the glenoid, Q: vertex of the inferior angle of the scapula, O: junction of the medial border of the scapula with the medial border of the scapular spine, S: is the point where the medial and lateral parts of the edge of the scapular spine meet. (b) Superior view of the scapula. Points O, S, and G are shown (G is covered by the acromion); Θ is defined as the angle between OS and OG.

 The study was conducted on 13 dry, intact adult scapulae (7 from the right and 6 from the left side of the body) of unknown sex procured from the University of Illinois at Chicago Anatomy Department collection. No existing pathologies or abnormalities of the scapulae were found except differences in size and the specimens were random. X-ray opaque lead balls 4 mm in diameter were mounted on each specimen with glue on points O, Q, and G. In addition a lead ball was placed on point S, which is the point where the medial and lateral parts of the edge of the scapular spine meet (Figure 1). This point is described in the literature [12] but is not named. The scapulae were then mounted in metal cages, using glue and plastic strings. The line OQ was kept vertical. The cages were numbered. 

An X-ray was taken of each specimen. The central ray went through the scapula from superior to inferior. A film in a 2 cm thick cassette was placed under the cage. The central ray of the X-ray ran vertically downward from a source 183 cm above the floor, passing through the specimen from superior to inferior. All X-rays were taken by an experienced technician.

 The positions of the lead balls were identified on the X-rays. The lead ball at point Q was easily identified because on the X-ray its image was smaller than the images of the other balls; Q was roughly 10 cm. closer to the X-ray cassette than the other points, so its image was smaller. 

### 2.2. Rationale

 The data are often obtained from recording devices that use vertical and horizontal coordinates. We first convert the coordinates to their equivalents based on the scapula. 

 Secondly we rotate the coordinate axes to positions OQ and the center line. Using cylindrical coordinates, we choose the third coordinate as the angle Θ between the center line (OG) and OS. In our study we measure this angle on the X-ray. In biomechanical studies we use the value of Θ determined by this and subsequent studies. We are interested only in the angle of the center line and we do not need the length of the center line as it runs to the humeral head.

 In summary, these points, O, Q, and S, are palpable, so their location can be established by light emitting diodes or other instruments. With Θ known, we can calculate the location of the center line; OG is perpendicular to OQ [3, 13].

### 2.3. Calculations

 We defined the point P as the point on the X-ray at which the perpendicular from S to line OG met line OG (Figure 1). PS and OP were measured on the X-ray 3 times and the means were obtained. Θ was obtained from the equation PS/OP = tan⁡Θ.

## 3. Results

Means measured for each specimen, OP and PS lengths, and Θ are shown in Table 1. The obtained Θ for the group was 28.74 ± 5.60°. 

## 4. Discussion

Knowing the position of glenoid is essential in analyzing force transmission from the humerus to the glenoid. However, to date we could find no study which determines the position of the glenoid from palpable points on the scapula. To obtain the glenoid position one must start with points on the scapula that are palpable. However, what we need is the position of the glenoid. Obtaining Θ would allow us to determine this position.

The study was conducted to test a method of finding the location of the glenoid using three palpable points on the scapula (O, Q, S). The Θ magnitude obtained in the current study allows calculation of the position of the glenoid using three palpable points (the inferior angle of the scapula (Q) and the junction between the scapular spine and the medial border of the scapula (O)) and the angle Θ between the OS line and the center line (that are obtained from X-rays). 

The obtained data on the position of glenoid could be used in calculation of forces across the shoulder joint. For example, to calculate the force vector from the humeral head across the shoulder, one can use a linked rigid body model. The obtained force and the angle of the glenoid and a critical angle, Θ, would allow calculating the amount of force transferred from the humeral head to the glenoid. 

There are some limitations that should be taken into consideration. First, following the literature [14, 15], we assumed that the body of the scapula is a plane, our plane OQG which might not be always true [16]. Second, we used the lead balls (4 mm in diameter); using smaller balls would allow more accurate measurements. The small number of specimens used in the current study allows us to obtain preliminary data only. Finally, the number of specimens needs to be larger to be able to apply the study outcome to individuals of different genders and ages.

 Nevertheless, the study outcome allows investigators to obtain the center line (OG-Figure 1(b)) from 3 palpable points. Furthermore, the direction of force from the humeral head can be compared with the direction of the center line in various positions, and normal positioning of the scapula and glenoid could be established. This could be applied to patients with certain conditions. Thus, in patients with dyskinesis, force direction from the humerus and glenoid could be found. Based on the obtained force direction, strengthening exercises could be prescribed so that normal force transmission can be restored. Also, in patients who need total shoulder replacement, the posterior part of the humeral head is very often worn away [2]. Appropriate exercises could be prescribed in the early stages of this condition, to redirect the force away from the abnormal part of the humeral head. It is also reported in the literature that overhead athletes (swimmers and pitcher in baseball) frequently develop shoulder problems. It is quite possible that these individuals develop dyskinesis and thus would benefit from appropriately designed physical therapy [5, 6].

## 5. Conclusions

The study outcome allows obtaining the position of the center line and as such the position of the glenoid from the positions of three palpable points on the scapula. This information could be used in calculation of forces across the shoulder joint, which in turn would allow optimizing the choice of strengthening exercises in patients with dyskinesis.

## Figures and Tables

**Table 1:** Values of Θ and the means of OP and PS.

Specimen #	OP (cm)	PS (cm)	Θ (deg)
5	2.7	1.4	1.3
6	3.0	2.0	1.9
7	3.43	1.59	0.18
8	4.36	2.02	0.63
9	3.78	2.13	1.48
10	5.64	2.44	0.83
11	4.19	1.81	2.18
12	4.69	3.19	0
13	2.67	1.47	1.4
14	10.94	4.67	2.2
15	4.87	2.78	1.38
17	3.81	3.42	2.33
